# Privacy Concerns and Configurational Patterns of Health Information Sharing Intention Among Patients with Chronic Diseases in Online Health Communities: An SEM and fsQCA Analysis

**DOI:** 10.3390/healthcare14193337

**Published:** 2026-10-06

**Authors:** Zongliang Wen, Xinting Qi, Yiheng Qin, Xin Ouyang, Yan Zhu

**Affiliations:** 1 School of Management, Xuzhou Medical University, Xuzhou 221004, China; wenzl@xzhmu.edu.cn (Z.W.); 202422050101@stu.xzhmu.edu.cn (X.Q.); qyh121501@163.com (Y.Q.); 302611311101@stu.xzhmu.edu.cn (X.O.); 2 School of Medical Information and Engineering, Xuzhou Medical University, Xuzhou 221004, China

**Keywords:** online health community, health information sharing intention, privacy concerns, patients with chronic diseases, structural equation modeling, fuzzy-set qualitative comparative analysis

## Abstract

**Highlights:**

**What are the main findings?**

- Privacy concerns were negatively associated with health information sharing intention, whereas attitude toward sharing, social rewards, past positive experience, and trust were positively associated with sharing intention.
- Low privacy concerns appeared in four of the five high-intention configurations. In the remaining minimized expression, privacy concerns were not retained and were therefore unrestricted; this result does not show that high privacy concerns promoted or were compensated in forming high intention.

**What are the implications of the main findings?**

- The findings identify privacy concerns, trust, and interaction experience as candidate considerations for future platform research, rather than demonstrating the effectiveness of specific platform features.
- Prospective studies should test whether privacy controls, verified information sources, and low-risk participation options affect actual disclosure behavior and patient outcomes.

**Abstract:**

Background/Objectives: Privacy concerns are negatively associated with health information sharing intention, but their role within combinations of facilitating conditions remains unclear. This study examined variable-level associations and configurational patterns among patients with chronic diseases. Methods: This single-center, cross-sectional study used a convenience sample of 428 patients who had used online health communities. The outcome was self-reported sharing intention rather than observed disclosure. Covariance-based structural equation modeling (SEM) assessed adjusted associations, and fuzzy-set qualitative comparative analysis (fsQCA) identified configurations sufficient for high or low intention under the selected calibration and thresholds. Results: The six-construct model was selected after data-dependent modification of an eight-construct candidate model and should therefore be interpreted as exploratory. Attitude toward sharing (β = 0.204), social rewards (β = 0.220), past positive experience (β = 0.220), and trust (β = 0.228) were positively associated with sharing intention (all *p* < 0.001), whereas privacy concerns were negatively associated with sharing intention (β = −0.119, *p* = 0.002). No single condition met the necessity threshold. The baseline fsQCA identified five high-intention and two low-intention configurations, although some expressions changed in sensitivity analyses. Low privacy concerns appeared in four high-intention configurations. In the remaining minimized expression, privacy concerns were not retained, so their level was unrestricted. The empirical truth table showed that high privacy concerns and high intention coexisted in some cases, but this does not establish a positive or compensatory contribution of privacy concerns. Conclusions: In this convenience sample, privacy concerns showed an overall negative association with self-reported sharing intention, while their role in minimized configurations varied with the analytical specification. The findings require independent, multicenter validation and do not establish intervention or platform-design effects.

## 1. Introduction

### 1.1. Background

Chronic-disease management extends across clinical care and daily life. Patients need to provide information about their medical history, symptoms, medication, and examinations over time. They also need to understand changes in their condition, adjust self-management, and cope with emotional and social effects [1]. Health information exchange therefore continues as patients gain knowledge, interpret their experiences, and seek support. Online health communities provide a digital space for patients to exchange disease knowledge, care experiences, and peer support outside formal clinical care [2,3,4].

Sharing health information in online health communities may provide several benefits. Describing personal experiences and health status can help patients ask specific questions and receive relevant responses. It may also help other patients in similar situations make decisions and develop coping plans [2,3,4,5,6,7]. However, medical history, medication, health status, psychological information, and identity information differ in sensitivity. Patients may face loss of control, identity exposure, or social judgment if their information is accessed by unintended parties. Similar risks arise when information is shared outside its original context or used without consent [5,6,8]. Health information sharing therefore involves both expected benefits and privacy costs.

This study focuses on health information sharing intention (HISI). HISI refers to a patient’s willingness to provide or exchange personal medical and health information with an online-health-community platform and other users. The study is limited to information exchange within the community, and the outcome variable measures behavioral intention. It does not cover the secondary use of health data by research institutions, businesses, or government agencies. Studies of third-party or secondary use further show that the purpose of data use and the identity of recipients matter in information sharing decisions [9,10,11].

Previous studies have generally reported a negative association between privacy concerns and willingness to disclose or share health information online. Information sharing decisions are also associated with outcome evaluation, social rewards, prior interactions, and judgments about the reliability of platforms and community members [6,8,12,13,14,15,16,17,18,19,20,21]. These findings position privacy concerns as an expected cost at the variable level. Whether low privacy concerns must appear in every sufficient configuration for high intention, however, remains a separate set-theoretic question.

Net-effect models estimate the adjusted association of each variable with sharing intention. A configurational analysis addresses a different question: whether distinct combinations of conditions are sufficient for high or low intention under specified calibration and minimization rules. We therefore compare the overall negative association of privacy concerns with their inclusion, absence, or omission in minimized sufficient expressions. Omission means that the condition is unrestricted within that expression; it does not indicate a positive, compensatory, or causal contribution.

### 1.2. Literature Review

The five retained antecedents represent related but non-equivalent judgments involved in health information sharing. Attitude toward sharing captures the overall evaluation of the behavior, drawing on the theory of planned behavior [22]. Social rewards represent anticipated recognition, reciprocity, and relational benefits, consistent with social exchange reasoning [7,15,16,20,21]. Privacy concerns represent expected loss of control or misuse costs in privacy calculus [6,8,14,18,19,23,24,25,26,27]. Past positive experience supplies retrospective evidence about prior interactions, whereas trust concerns the perceived reliability of the platform and community members. Together, these constructs organize behavioral evaluation, expected benefits, privacy costs, experiential feedback, and relationship reliability within one empirical framework.

These constructs can overlap and may follow competing causal orders. Past positive experience may inform trust, expected rewards, or attitude; trust may be associated with lower privacy concerns; and attitude may partly summarize evaluations captured by the other constructs. The present cross-sectional model treats them as parallel correlates so that their mutually adjusted associations and configurations can be compared. It does not establish a unified causal sequence, mediation structure, or temporal ordering. Longitudinal or experimental work is required to distinguish these alternatives.

Risk and relationship factors form the other side of information sharing decisions. Privacy concerns reflect worries that personal health information may be misused, accessed by unintended parties, or used beyond the patient’s control. According to privacy calculus, these expected outcomes increase the perceived cost of sharing and may reduce sharing intention [6,8,14,18,19,23,24,25,26,27]. Trust reflects judgments about the reliability of relevant actors in the information sharing environment. In this study, trust includes confidence in the services, rules, and information processing of online-health-community platforms. It also includes confidence in the information and commitments provided by other community members, but not trust in medical staff. Previous studies have linked trust in health websites, platforms, or community members with stronger disclosure or sharing intention [13,14,17,18,28,29,30].

Earlier studies established variable-level relationships among attitudes, benefits, experience, privacy concerns, trust, and information sharing decisions [6]. Xiang et al. [13] had already combined structural equation modeling (SEM) and fuzzy-set qualitative comparative analysis (fsQCA) to examine perceived ease of use, perceived usefulness, perceived behavioral control, attitude, and trust in relation to online health information sharing. Accordingly, the present study does not claim that combining SEM with fsQCA, adding one variable, or using a chronic-disease sample is itself a methodological or conceptual innovation.

The incremental proposition examined here is narrower: an overall negative association between privacy concerns and sharing intention does not necessarily imply that low privacy concerns are required in every minimized sufficient expression for high intention. We therefore compare the variable-level association of privacy concerns with their configurational role across high and low outcomes, report all equally parsimonious models, and examine solution sensitivity. The chronic-disease sample provides a context of repeated exchange of potentially sensitive information, but the absence of a comparison group prevents claims that the observed pattern is unique to patients with chronic diseases. The contribution is therefore an exploratory refinement of the boundary conditions of privacy costs, not a new general theory.

### 1.3. Theoretical Framework and Research Hypotheses

#### 1.3.1. Attitude Toward Sharing

Attitude toward sharing (ATT) is a patient’s overall evaluation of whether sharing personal health information in an online health community is beneficial, appropriate, and worthwhile. The theory of planned behavior states that a positive evaluation of a behavior’s consequences increases the intention to perform that behavior [22]. Patients with chronic diseases are more likely to view sharing as beneficial when they believe it can solve problems, help others, or improve community interaction. Research on online health communities also places attitude toward sharing at the center of the formation of sharing intention [13,15,17,20]. We therefore propose the following hypothesis:

**H1.**  *Attitude toward sharing is positively associated with patients with chronic diseases’ health information sharing intention.*

#### 1.3.2. Social Rewards

Social rewards (SR) are the relational and psychological benefits that patients expect from sharing. These benefits include receiving responses and recognition, maintaining supportive relationships, and gaining satisfaction from helping others. Social exchange theory suggests that people decide whether to share information by considering expected benefits and personal costs. When sharing provides social benefits, the value of participation increases [7,15,16,20,21]. Patients with chronic diseases may face similar management problems over time. Responses and confirmation from community members can therefore support continued participation. In this study, social rewards refer to expected recognition, social connection, and psychological benefits. They are considered separately from social support or benefits that have already been received.

**H2.**  *Social rewards are positively associated with patients with chronic diseases’ health information sharing intention.*

#### 1.3.3. Past Positive Experience

Past positive experience (PPE) refers to previous experiences of receiving useful advice, practical help, and positive responses from health websites or online health communities. Past interactions provide information for judging the value of future sharing. More positive experiences may reduce uncertainty about sharing again and make expected benefits clearer [3,4,7,14,15]. This expectation is important in chronic-disease management, which often requires repeated information seeking and changes in self-management. In this study, past positive experience, attitude toward sharing, and trust have different meanings. They refer to past benefits, current evaluation of sharing behavior, and judgments about reliability, respectively.

**H3.**  *Past positive experience is positively associated with patients with chronic diseases’ health information sharing intention.*

#### 1.3.4. Privacy Concerns

Privacy concerns (PC) are patients’ worries that their health information may be misused, accessed by unintended parties, shared without consent, or used beyond their control. Privacy calculus suggests that people compare expected benefits with possible privacy costs when deciding whether to disclose information. Sharing is usually less acceptable when expected risk is higher [8,14,18,19,23,24,25]. Patients with chronic diseases may need to discuss medical history, medication, health status, and psychological information. If this information is shared beyond the expected scope, it may expose identity or lead to negative social judgment [5,6,8,26,27]. Privacy concerns may therefore reduce sharing intention even when sharing could provide benefits.

**H4.**  *Privacy concerns are negatively associated with patients with chronic diseases’ health information sharing intention.*

#### 1.3.5. Trust

Trust (TR) is a patient’s overall judgment of the reliability of an online-health-community platform and its members. For the platform, trust concerns the reliability of services, rules, information processing, and privacy protection. For community members, trust concerns the information they provide, the commitments they make during interactions, and their sharing behavior [13,17,18,28,29,30]. Trust can reduce uncertainty about misuse, incorrect information, and failures in platform rules. It may therefore increase the acceptability of the consequences of sharing [14,28]. This study models platform trust and member trust as one overall construct. It focuses on online health communities and does not include trust in medical staff.

**H5.**  *Trust is positively associated with patients with chronic diseases’ health information sharing intention.*

### 1.4. Research Model and Research Questions

The research model used HISI as the outcome. H1-H5 described the theory-informed directions of association for ATT, SR, PPE, PC, and TR. RQ1 and RQ2 addressed sufficient configurations for high and low intention and configurational asymmetry. Additional exploratory interaction analyses were conducted after the primary SEM and fsQCA analyses. The specific models, contextual comparisons, disease-group contrasts, and multiplicity families were not preregistered or prospectively prespecified and are therefore treated as post hoc exploratory analyses rather than confirmatory tests. Table 1 summarizes the correspondence between the theoretical propositions and empirical tests.

RQ1: Which combinations of conditions are sufficient for high health information sharing intention under the selected calibration and analytical thresholds?

RQ2: Which combinations of conditions are sufficient for low health information sharing intention, and how do they differ from the high-intention configurations?

Post hoc exploratory question: Does the association between privacy concerns and sharing intention vary across facilitating conditions, information sensitivity, privacy sensitivity, or disease type?

## 2. Materials and Methods

### 2.1. Research Design

A single-center, cross-sectional questionnaire survey was conducted from 21 to 26 July 2025 using convenience sampling at Nantong Sixth People’s Hospital, Jiangsu Province, China. Recruitment took place in outpatient waiting areas and inpatient wards, coordinated on-site with the hospital’s Public Health Department and participating clinical departments. Participants were eligible if they were aged 18 years or older, had at least one chronic disease confirmed by the relevant clinical department, had previously used an online health community, were able to understand and complete the questionnaire, and had voluntarily agreed to participate. Individuals who did not meet these eligibility criteria or declined consent were not enrolled. Participants received the study information and provided informed consent before completing the questionnaire. Investigators reconfirmed their willingness to participate before questionnaire completion. After questionnaire return, records were excluded if respondents had stopped partway through with substantial missingness, defined as more than half the items in any core construct or more than 20% of all questionnaire items unanswered, or if respondents stated during the survey that they could not reliably recall relevant online-health-community experiences. Of 468 returned questionnaires, 11 were excluded for substantial missingness and 29 for unreliable recall. No questionnaire was excluded solely for logically inconsistent answers. The final analytical sample comprised 428 participants. Before participant recruitment, the target sample size was determined pragmatically with reference to the complexity of the initial SEM specification. The candidate model comprised eight latent constructs measured by 28 observed indicators. Schumacker and Lomax [31] discuss commonly cited heuristic guidance in SEM, including approximately 10–20 cases per observed variable, while also noting that sample requirements depend on model complexity and other design characteristics. Applying this heuristic to the 28 observed indicators yielded a broad reference range of approximately 280–560 cases. We therefore set a pragmatic target of approximately 400 valid responses, corresponding to about 14 cases per observed indicator and lying within this reference range. Allowing for an anticipated invalid-response rate of approximately 15%, the recruitment target was approximately 471 returned questionnaires. A total of 468 questionnaires were returned, of which 428 were retained for analysis, exceeding the target of 400 valid responses. This planning approach was heuristic and model-complexity-based rather than a formal effect-size-based power analysis. No fixed minimum-sample rule was applied to fsQCA. With five antecedent conditions, 32 logically possible truth-table configurations were evaluated; the baseline case-frequency threshold was 5, and a threshold of 10 was examined in sensitivity analysis. The study was approved by the Ethics Committee of Xuzhou Medical University on 15 July 2025 (approval no. XZHMU-25S07151B), before the approved study start date of 20 July 2025.

### 2.2. Questionnaire and Variable Measurement

The formal survey used a Chinese-language questionnaire covering online-health-community use, demographic and disease characteristics, and the study constructs. Items were drawn from published instruments and adapted where needed for the online-health-community and chronic-disease context; Supplementary File S1 provides English renderings of all 28 candidate items and identifies their sources or adaptation basis. Because no verifiable record of a formal forward–back translation procedure was available, linguistic and cross-language measurement equivalence could not be independently verified, although readability and comprehension were assessed in the pilot test. All retained construct items used a seven-point Likert scale, were scored in the same direction, and were averaged within each construct. A 35-participant pilot test was conducted on 20 July 2025 within the scope of the ethics approval. All pilot participants received the study information and provided informed consent. Their feedback was used to assess readability, item comprehension, and completion flow; they were not included among the 468 formal questionnaires or the final 428-case analytical sample.

The candidate measurement model contained eight reflective constructs: HISI, ATT, perceived behavioral control (PBC), SR, PPE, PC, social support (SS), and TR. The eight-factor confirmatory factor analysis (CFA) model had good global fit, but PBC, SS, and TR showed severe local overlap. Their latent correlations were 0.991 for PBC–TR, 0.987 for SS–TR, and 0.974 for PBC–SS; the corresponding heterotrait–monotrait ratio (HTMT) values were 0.993, 0.988, and 0.976. In the eight-construct structural model, the standardized coefficients were −2.499 for PBC, −0.784 for SS, and 3.469 for TR, with large standard errors. After inspecting these results and item content, we retained TR because reliability judgments were most directly aligned with the privacy-focused research question, and removed PBC and SS from the revised main model. This decision was data-dependent. The candidate eight-construct specification and its corresponding structural paths are reported as part of the model-development record. Because no contemporaneous documentation was identified for separate directional hypotheses concerning PBC and SS, these paths are not presented as prespecified hypotheses. The six-construct model and its path estimates are therefore exploratory and require validation in an independent sample. Supplementary File S2 provides descriptive composites and key HTMT diagnostics; Supplementary Data S2 (SEM Model) reports the complete eight-factor and six-factor latent-correlation matrices, model estimates, and same-sample alternatives. Table 2 summarizes the retained constructs and their operational measurements.

### 2.3. Data Analysis

#### 2.3.1. Descriptive Statistics, Measurement Quality and Common Method Risks

The analysis first described the sample and reported the means and standard deviations of the main variables. Internal consistency was assessed with Cronbach’s alpha and composite reliability (CR) [32]. Convergent validity was assessed with standardized factor loadings and average variance extracted (AVE) [33]. Discriminant validity was assessed with the heterotrait–monotrait ratio (HTMT) [34]. Multicollinearity was examined with variance inflation factors (VIFs) based on the construct composite scores. The reference thresholds were at least 0.70 for alpha and CR, at least 0.50 for AVE, and below 0.85 for HTMT [32,33,34].

We fitted a 28-item candidate-set one-factor diagnostic model, the original eight-factor diagnostic model, and the revised six-factor model. The one-factor diagnostic was reported only as a common-method diagnostic; its global fit was not directly compared with that of the 21-item revised six-factor model. These analyses were used to assess whether one factor explained the candidate-set covariance structure and to identify construct overlap. Poor one-factor fit does not exclude common-method bias because all predictors and the outcome were self-reported by the same participant in one survey [35].

#### 2.3.2. Latent Variable Structural Equation Modeling

The revision analyses fitted the six-construct covariance-based CFA and SEM in Python 3.12.14 using semopy 2.3.11 with the MLW objective and SLSQP optimization. HISI was regressed on ATT, SR, PPE, PC, and TR, while the five predictors were allowed to co-vary. Sandwich covariance estimates provided heteroscedasticity-robust standard errors for unstandardized parameters; two-sided 95% confidence intervals used a normal approximation. Standardized coefficients were reported separately. The fit indices were conventional, not robustly scaled, and were interpreted with reference to established cautions regarding universal cutoffs [36,37]. Because the structural specification reparameterized the freely estimated covariances between HISI and all five predictors without adding covariance restrictions, the six-factor CFA and structural model had the same degrees of freedom and numerically equivalent fit. Repeated fit indices were not interpreted as independent confirmation of path direction. Cross-sectional paths were interpreted as adjusted associations, not causal effects.

#### 2.3.3. fsQCA

For fsQCA, construct means were directly calibrated into membership scores. Because every construct used the same seven-point response format, scores of 2, 4, and 6 represented substantive disagreement, maximum ambiguity or neutrality, and substantive agreement, respectively, and served as full non-membership, crossover, and full-membership anchors. Exact memberships of 0.500 were shifted uniformly to 0.501 to permit truth-table assignment. This affected 27 HISI, 23 ATT, 41 SR, 37 PPE, 15 PC, and 15 TR observations. Low-condition sets were calculated as one minus the corresponding membership score. Quantile-based anchors were examined as a sensitivity analysis because common response semantics do not imply identical empirical distributions across constructs.

Necessity was evaluated with a consistency threshold of 0.90. Baseline truth tables used a case-frequency threshold of 5, raw consistency of 0.80, and a proportional reduction in inconsistency (PRI) threshold of 0.70. The frequency threshold limited reliance on sparse rows while retaining empirical coverage across the 32 possible combinations in 428 cases. Consistency and PRI thresholds were analytical settings, not uniquely determined cutoffs. The calibration, truth-table, and minimization decisions followed established fsQCA guidance [38,39,40,41,42]. Supplementary File S3 contains the complete baseline truth table. Supplementary Data S1 (fsQCA) reports calibration rules, crossover handling, contradictory rows, logical remainders, directional expectations, complex and intermediate solutions, all equally parsimonious solutions, and sensitivity results. Case-level membership scores are not included in public Supplementary Materials because the underlying health-questionnaire records remain subject to consent, ethics, and hospital data-governance restrictions.

Theory-informed directional expectations for the intermediate solution specified high ATT, SR, PPE, and TR and low PC for high HISI, with the reverse pattern for low HISI. These expectations were not preregistered. Complex solutions excluded logical remainders, parsimonious solutions allowed all remainders, and intermediate solutions allowed only remainders consistent with these expectations. Under the baseline frequency threshold, 22 configurations for each outcome were logical remainders and none was used as an easy counterfactual; the complex and intermediate solutions were therefore identical.

#### 2.3.4. Exploratory Interaction Analysis Methods

Post hoc exploratory linear models used standardized composite scores and added one PC interaction at a time to the five main effects. The approved protocol permitted exploratory interaction and robustness analyses; however, the particular models, interaction terms, subgroup contrasts, and multiplicity families reported here were not preregistered or prospectively prespecified and were finalized after the primary SEM and fsQCA analyses. The five facilitating-condition interactions were PC × TR, PC × ATT, PC × SR, PC × PPE, and PC × a composite of standardized ATT, SR, PPE, and TR scores. Models used HC3 heteroscedasticity-robust standard errors and reported unstandardized coefficients, 95% confidence intervals, and unadjusted *p* values. Benjamini–Hochberg correction was applied to this five-test exploratory family. Separate contextual analyses examined PC × information sensitivity, PC × privacy sensitivity, and a four-degree-of-freedom PC × disease-type interaction; these three tests formed a contextual family. As a sensitivity analysis, all eight interaction tests were pooled into one BH family. Four disease-group contrasts against the cardiovascular reference group formed a separate BH family. Within each family, unadjusted *p* values were sorted in ascending order and the BH-adjusted q value for rank i was the minimum of m × p(j)/j across ranks j ≥ i, capped at 1, where m is the number of tests in that family. Disease coding was cardiovascular disease (reference; *n* = 189), diabetes (*n* = 117), chronic obstructive pulmonary disease (*n* = 85), cancer (*n* = 34), and other disease (*n* = 3). For these analyses, disease type was represented by one recorded category per participant; coexisting conditions were not modeled separately. These analyses were secondary and hypothesis generating. They were conducted with statsmodels 0.15.0 in Python 3.12.14. Supplementary File S4 presents the main exploratory results; Supplementary Data S3 (Exploratory) provides model coefficients, conditional slopes, disease-group comparisons, and multiplicity adjustments.

#### 2.3.5. Robustness and Data Integrity Diagnosis

SEM sensitivity analyses compared conventional and sandwich-robust standard errors and refitted models after separately removing PC3, HISI2, and TR4, followed by a specification excluding all three items. These items were selected for item-deletion sensitivity analyses because their wording showed comparatively greater potential for construct-boundary ambiguity. These analyses used the same sample and did not constitute independent validation. Item-level skewness ranged from −0.749 to 0.661 and excess kurtosis from −0.710 to 0.097. These univariate diagnostics do not establish multivariate normality; sandwich-robust standard errors were therefore reported alongside conventional estimates. The final 428-case dataset had no missing values in the 21 main-model items, so no imputation or full-information missing-data procedure was used for the main CFA or SEM. The 40 exclusions from the 468 collected questionnaires were handled before creation of the analytical dataset, as described in Section 2.1.

fsQCA sensitivity analyses varied the frequency threshold, raw-consistency threshold, PRI threshold, calibration anchors, exact-crossover handling and selected item composition. In addition to the baseline 0.500-to-0.501 rule, exact-crossover scores were shifted to 0.499; a stricter PRI cutoff of 0.75 was also tested. We compared truth-table selection, configuration expressions, the number of equally parsimonious solutions, consistency, PRI, and coverage. The revision analyses used a documented custom minimization workflow implemented in Python 3.12.14. Supplementary File S3 and Supplementary Data S1 (fsQCA) provide the settings, truth tables, solutions, and sensitivity results; neither includes case-level membership scores.

## 3. Results

### 3.1. Sample Characteristics

The final sample included 428 patients with chronic diseases who had used online health communities. There were 219 men (51.2%) and 209 women (48.8%). The 41–60-year age group was the largest (39.7%), followed by participants older than 60 years (27.1%). A total of 60.7% had used the Internet for at least 10 years, and 36.0% had used online health communities for 4–5 years. The largest illness-duration group was 5–9 years (41.1%). The main disease types were cardiovascular disease (44.2%) and diabetes (27.3%). Table 3 presents the full demographic and disease characteristics.

### 3.2. Descriptive Statistics and Measurement Model

The mean (SD) of HISI was 4.623 (1.515). The corresponding means for ATT, SR, PPE, and TR were 4.848, 4.818, 4.742, and 4.887, and the mean for PC was 3.274. Across the six constructs, Cronbach’s alpha and CR ranged from 0.897 to 0.927, AVE ranged from 0.712 to 0.771, and all standardized loadings exceeded 0.80. Table 4 reports the descriptive statistics, reliability, and convergent validity results.

The revised six-factor measurement model fitted the sample covariance structure well. The 28-item candidate-set one-factor diagnostic model fitted poorly, but this result does not rule out common-method bias [35] and is not directly comparable with the 21-item revised six-factor model. The original eight-factor model also had good global fit; nevertheless, the PBC-TR, SS-TR, and PBC-SS latent correlations were 0.991, 0.987, and 0.974, and the corresponding HTMT values were 0.993, 0.988, and 0.976. These local diagnostics motivated the data-dependent six-construct revision described in Section 2.2. In the revised model, all HTMT values were below 0.85 (maximum 0.625), and composite-score VIFs were below 2.0 (maximum 1.760). The six-factor model should be interpreted as exploratory because the same sample informed model selection and estimation. Model-fit indices are reported in Table 5.

### 3.3. Structural-Model Results

The revised structural model explained 56.6% of the variance in HISI. Adjusted associations were positive for ATT (β = 0.204, *p* < 0.001), SR (β = 0.220, *p* < 0.001), PPE (β = 0.220, *p* < 0.001), and TR (β = 0.228, *p* < 0.001), and negative for PC (β = −0.119, *p* = 0.002). Robust 95% confidence intervals for the unstandardized coefficients excluded zero for all five paths (Table 6). These directions were consistent with H1–H5, but the results do not establish temporal or causal direction, and the similar positive coefficients should not be ranked strictly by magnitude. Figure 1 visualizes the standardized path coefficients.

### 3.4. fsQCA Results

#### 3.4.1. Necessary Conditions

No single condition achieved a consistency of 0.90, so no necessary condition was found. For high HISI, the consistencies of TR, ATT and SR were 0.898, 0.897 and 0.894 respectively, all close to but not reaching the necessity threshold; the consistencies of PPE and low PC were 0.878 and 0.797 respectively. Therefore, no individual condition met the conventional necessity criterion for high HISI.

#### 3.4.2. Full Configuration of High and Low Sharing Intention

Under the baseline specification, the intermediate and complex solutions for high HISI were identical and contained five configurations. HC1 was SR × PPE × ~PC × TR; HC2 was ATT × PPE × ~PC × TR; HC3 was ATT × SR × ~PC × TR; HC4 was ATT × SR × PPE × TR; and HC5 was ATT × SR × PPE × ~PC. Low PC appeared in four configurations. PC was omitted from HC4, meaning that its level was unrestricted in that minimized expression. This omission does not indicate that high PC promoted high intention or that the other conditions compensated for PC. Three equally parsimonious models existed, and core or peripheral status varied across them; only the cross-model invariant conditions are reported in Table 7.

**Table 7:** Intermediate solutions of fsQCA for high and low health information sharing intention.

Path	Configuration Expression	Cross-Model Invariant Core	Other Configuration Conditions	Consistency/PRI	Raw/Unique Coverage
HC1	SR × PPE × ~PC × TR	~PC	SR, PPE, TR	0.920/0.893	0.646/0.025
HC2	ATT × PPE × ~PC × TR	ATT	PPE, ~PC, TR	0.916/0.888	0.644/0.023
HC3	ATT × SR × ~PC × TR	ATT	SR, ~PC, TR	0.917/0.889	0.658/0.037
HC4	ATT × SR × PPE × TR	ATT	SR, PPE, TR	0.925/0.903	0.737/0.116
HC5	ATT × SR × PPE × ~PC	ATT	SR, PPE, ~PC	0.930/0.907	0.648/0.026
LC1	~ATT × ~PPE × PC × ~TR	none	~ATT, ~PPE, PC, ~TR	0.936/0.838	0.284/0.028
LC2	~ATT × ~SR × ~PPE × ~TR	none	~ATT, ~SR, ~PPE, ~TR	0.941/0.891	0.401/0.145

Note: ~ means the condition is absent. There are three equally parsimonious models for high and low HISI, and only the conditions that remain unchanged across P1–P3 are listed as invariant cores in the table. High HISI overall solution consistency = 0.877, PRI = 0.841, overall coverage = 0.849, and total unique coverage = 0.227; low HISI overall solution consistency = 0.933, PRI = 0.879, overall coverage = 0.429, and total unique coverage = 0.173. Figure 2 presents the configurational matrix for the high- and low-intention solutions.

**Figure 2:**
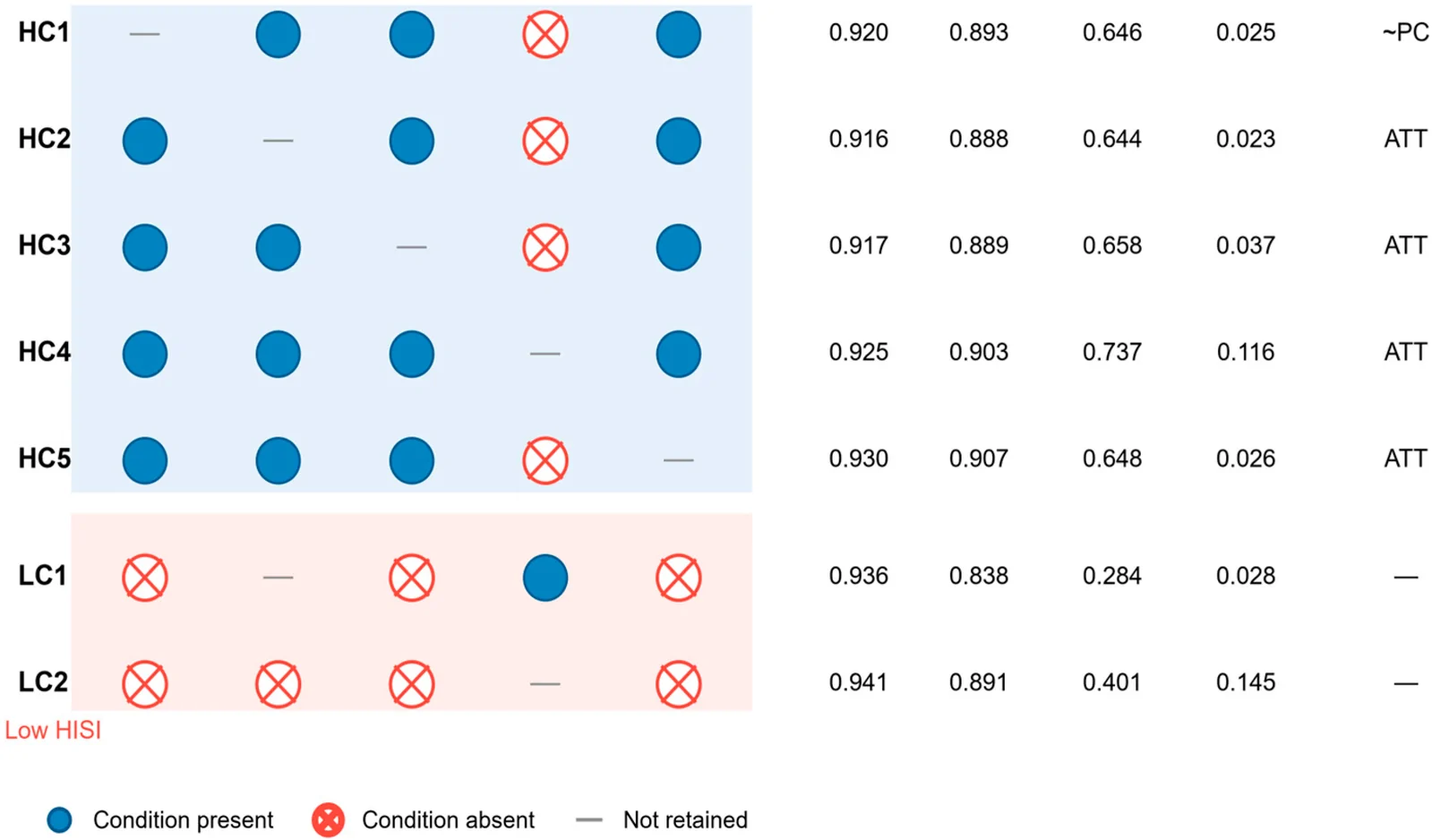
fsQCA configurational matrix of high and low health information sharing intention. Solid circles indicate that the condition exists, open crossed circles indicate that the condition is absent, and dashes indicate that a condition was not retained. The right-side lists consistency, PRI, raw coverage, unique coverage, and core conditions that remain unchanged across P1–P3. Because the core–peripheral classification varies across the three equally parsimonious models, circle size is not used to distinguish core from peripheral conditions.

The baseline intermediate solution for low HISI contained two configurations: LC1 (~ATT × ~PPE × PC × ~TR) and LC2 (~ATT × ~SR × ~PPE × ~TR). Three equally parsimonious models were available, and no core condition remained invariant across them. The specific low-intention expressions also changed under several threshold, calibration, and item-composition checks. They are therefore exploratory results under the baseline specification, not stable or unique pathways.

The full baseline truth table included six selected high-outcome rows and three selected low-outcome rows. The 11111 row represented high ATT, SR, PPE, PC, and TR and included 63 cases (consistency 0.914; PRI 0.853). This row documents empirical coexistence of high PC and high HISI in some cases. It does not establish that high PC contributed to the sufficient configuration. When the adjacent 11111 and 11101 rows entered minimization together, PC was omitted from HC4 and was therefore unrestricted in the minimized expression. Supplementary File S3 reports the complete truth tables; Supplementary Data S1 (fsQCA) separately reports all minimized solutions and sensitivity results.

### 3.5. Exploratory Interaction Analysis Results

None of the five facilitating-condition interactions met q < 0.05 after Benjamini–Hochberg correction. PC × TR (b = 0.072, 95% CI [0.010, 0.134], unadjusted *p* = 0.022, q = 0.055), PC × ATT (b = 0.021, 95% CI [−0.038, 0.080], *p* = 0.477, q = 0.477), PC × SR (b = 0.030, 95% CI [−0.026, 0.085], *p* = 0.296, q = 0.370), PC × PPE (b = 0.076, 95% CI [0.015, 0.137], *p* = 0.015, q = 0.055), and PC × the facilitating-condition composite (b = 0.068, 95% CI [0.002, 0.134], *p* = 0.044, q = 0.073) did not provide multiplicity-adjusted evidence of moderation.

In the three-test contextual family, PC × information sensitivity had b = −0.014 (95% CI [−0.091, 0.063], unadjusted *p* = 0.721, q = 0.721), PC × privacy sensitivity had b = −0.047 (95% CI [−0.124, 0.030], *p* = 0.231, q = 0.346), and the omnibus PC × disease-type interaction had Wald χ^2^(4) = 6.229 (*p* = 0.183, q = 0.346). When all eight interaction tests were pooled as a sensitivity family, the minimum q was 0.088 (PC × PPE and PC × TR); none met q < 0.05.

Using cardiovascular disease as the reference group (*n* = 189), the PC-slope contrasts were: diabetes (*n* = 117), b = −0.003, 95% CI [−0.173, 0.167], unadjusted *p* = 0.976, q = 0.976; chronic obstructive pulmonary disease (*n* = 85), b = 0.013, 95% CI [−0.161, 0.186], *p* = 0.888, q = 0.976; cancer (*n* = 34), b = −0.244, 95% CI [−0.510, 0.021], *p* = 0.071, q = 0.280; and other diseases (*n* = 3), b = 0.532, 95% CI [−0.174, 1.239], *p* = 0.140, q = 0.280. These four q values were adjusted as a separate family. The highly unequal group sizes, particularly *n* = 3 in the last group, limit inference; nonsignificance does not establish equivalence. Conditional slopes and full model coefficients are provided in Supplementary File S4 and Supplementary Data S3 (Exploratory).

### 3.6. Robustness Checks

After Huber–White robust standard errors were used, the direction and significance of all five SEM paths remained unchanged. ATT, SR, PPE, and TR remained positively associated with HISI, with *p* < 0.001 for each path. PC remained negatively associated with HISI (*p* = 0.002). The robust standard errors were slightly larger than the standard maximum-likelihood estimates. For example, the standard error for TR increased from 0.069 to 0.083. The confidence intervals for all five paths remained above or below zero.

In the three alternative measurement specifications, PC3, HISI2, and TR4 were removed separately. Model fit ranged from 1.148 to 1.228 for chi-square divided by degrees of freedom, from 0.995 to 0.997 for CFI, from 0.994 to 0.996 for TLI, and from 0.019 to 0.023 for RMSEA. The standardized coefficients ranged from 0.200 to 0.205 for ATT, 0.219 to 0.260 for SR, 0.194 to 0.217 for PPE, and 0.219 to 0.227 for TR. The standardized coefficient for PC ranged from −0.126 to −0.093. All path directions were consistent with the main model, and all *p* values were below 0.05. After HISI2 was removed, the PC path decreased to β = −0.093 (*p* = 0.020). The PC coefficient was β = −0.126 after PC3 was removed (*p* = 0.001) and β = −0.117 after TR4 was removed (*p* = 0.002). The variance explained in HISI ranged from R^2^ = 0.559 to 0.568 across the three specifications. When PC3, HISI2, and TR4 were removed together, the five standardized coefficients were 0.201, 0.263, 0.189, −0.101, and 0.219. The R^2^ of HISI was 0.561, and all path directions remained consistent with the main model.

The fsQCA results depended on some analytical settings. Raising raw consistency from 0.80 to 0.85 did not change the five high- and two low-intention baseline configurations. Raising frequency from 5 to 10 reduced the high-intention configurations from five to four and the low-intention configurations from two to one. Lowering PRI to 0.65 yielded four high- and two low-intention configurations, whereas raising PRI to 0.75 retained all five high-intention configurations but reduced the low-intention solution from two configurations to one. Shifting exact-crossover memberships from 0.500 to 0.499 reproduced the same five high- and two low-intention expressions; changes in overall solution consistency and coverage were less than 0.001. Quantile calibration yielded five high- and one low-intention configuration and reduced high-outcome coverage from 0.849 to 0.702. Item-composition checks also changed the low-intention solution. Both outcomes had three equally parsimonious baseline models. Accordingly, the reported configurations are specification-dependent sufficient expressions, particularly for low intention, rather than stable or unique pathways.

## 4. Discussion

### 4.1. Main Findings

In the revised six-construct model, privacy concerns were negatively associated with sharing intention after adjustment for the other constructs. ATT, SR, PPE, and TR were positively associated with sharing intention. These sample-specific associations are broadly consistent with earlier online-health-community studies [6,8,12,13,14,15,16,17,18,19,20,21,28], but the model was selected after data-dependent construct removal and all variables were measured concurrently by self-report. The findings therefore do not establish that any construct preceded or caused sharing intention.

The baseline fsQCA provided a distinct set-theoretic result. Low PC appeared in four of five high-intention expressions, while PC was omitted from HC4 and was therefore unrestricted in that expression. The 11111 truth-table row showed empirical coexistence of high PC and high HISI in 63 cases. Neither result shows that high PC promoted intention or that ATT, SR, PPE, and TR compensated for privacy concerns. This distinction between empirical truth-table observations and minimized sufficient expressions is central to interpreting the configurational findings.

The two-baseline low-intention expressions both contained low PPE and low TR, but their core status was not invariant across three equally parsimonious models, and the solution changed under several sensitivity settings. The high- and low-intention results illustrate configurational asymmetry within the baseline analysis, but they should not be treated as unique, stable, or causal pathways. Independent samples and prespecified analytical choices are needed to assess reproducibility.

The post hoc interaction analyses did not provide adequate evidence of moderation after multiplicity correction. The two smallest q values were 0.055 in the five-test exploratory facilitating-condition family and 0.088 in the pooled eight-test sensitivity family; the disease-type interaction was also nonsignificant. Nominally nonzero conditional slopes at selected moderator values describe model-based conditional associations only. They do not establish a significant interaction when the interaction term does not survive multiplicity correction. These findings do not confirm buffering, compensation, or disease-group differences and are retained only as transparent exploratory results.

### 4.2. Theoretical Implications

The theoretical contribution is an incremental and exploratory refinement of privacy calculus. The negative adjusted SEM coefficient describes the variable-level association of privacy concerns with intention. The fsQCA shows that low PC was not retained in every minimized sufficient expression under the baseline specification. These statements concern different analytical levels. Omission from HC4 means that PC was unrestricted there, not that high PC had a beneficial effect or was offset by other conditions.

The study also places behavioral evaluation, anticipated social rewards, prior experience, privacy costs, and reliability judgments in a shared empirical framework. This comparison does not establish a single integrated causal theory. ATT may partly summarize evaluations informed by rewards, experience, trust, and privacy concerns; PPE may precede trust or attitude; and trust may be related to PC. The cross-sectional design cannot distinguish between these competing sequences. Because PBC was removed after model diagnostics, the revised model is not a complete test or extension of the theory of planned behavior.

For configurational research, the study explicitly examines privacy risk across both high and low intention and reports multiple equally parsimonious models and sensitivity checks. Equifinality and asymmetry are established fsQCA principles rather than novel propositions introduced here. The contribution lies in applying those principles to the conditional role of privacy concerns in a chronic-disease sample, with clear limits on generalizability and stability.

### 4.3. Practical Implications

The findings identify privacy concerns, trust, attitude, anticipated social rewards, and prior experience as factors that platform researchers may consider together. However, the study did not observe actual disclosure, manipulate platform features, compare interventions, or measure patient outcomes. It therefore cannot determine whether changing any platform feature would alter sharing behavior, information security, or health outcomes.

Access controls, verified information sources, withdrawal procedures, staged disclosure, response monitoring, and tailored participation routes should be treated as design hypotheses for prospective evaluation. Future preregistered experiments, field trials, or longitudinal studies could test their acceptability and effects on actual disclosure, withdrawal, sustained use, safety events, and patient-reported outcomes. Until such evidence is available, these features should not be presented as interventions established by the present results.

### 4.4. Limitations and Future Research

This study has several limitations. The prospective sample-size target was based on commonly used heuristic SEM rules and model complexity rather than a model-specific Monte Carlo or effect-size-based power analysis; therefore, the adequacy of the sample should also be judged from estimation precision, confidence intervals, model stability, and the sensitivity analyses reported here. First, its single-center convenience sample may be affected by selection and self-selection and cannot represent all patients with chronic diseases. Regional and institutional differences in digital-health access, health literacy, privacy norms, social relationships, data-governance practices, patient mix, and institutional trust may alter privacy evaluations and sharing intentions. Multicenter probability or stratified samples are needed. Second, the original eight-construct model was revised after diagnostics showed overlap and unstable estimates among PBC, SS, and TR. The PBC items also mixed personal control capability, risk reduction, and platform privacy-measure content; this breadth blurred the construct boundary and limits substantive interpretation of the removed PBC factor. The six-construct model is therefore data-dependent and may overstate fit or path stability in this sample. Some item wording, particularly HISI2 and PC3, may not capture the intended construct boundaries as narrowly as is ideal, although item-deletion sensitivity analyses did not materially alter the main interpretation. Third, all predictors and the outcome were self-reported in one survey, creating risks from common-method variance, social desirability, recall, and consistency responding; poor one-factor fit does not remove these risks. Because no verifiable record of a formal forward–back translation procedure was available, linguistic and cross-language measurement equivalence could not be independently verified. Fourth, cross-sectional data cannot establish temporal order or exclude reverse or reciprocal relationships. Fifth, HISI measures intention rather than observed disclosure. Sixth, the combined TR construct may mask differences between platform and member trust and may overlap with experience, support, and control. Seventh, no exploratory interaction remained significant after multiplicity correction. Disease groups were markedly unequal, and the single disease-category variable did not capture multimorbidity; nonsignificant comparisons do not demonstrate equivalence. Finally, the fsQCA expressions, particularly for low intention, varied across thresholds, calibration, item composition, and equally parsimonious models.

Future research should validate the measurement and structural models in independent, multicenter samples before testing mediation or temporal sequences. Longitudinal studies, platform logs, cross-lagged designs, and preregistered experiments could separate intention from actual behavior and evaluate causal ordering. Separate measures of platform trust, member trust, PBC, and social support would clarify construct boundaries. Configurational studies should preregister calibration, thresholds, directional expectations, and the treatment of multiple parsimonious solutions. Adequately powered disease-group samples are needed before drawing conclusions about heterogeneity.

## 5. Conclusions

In this single-center convenience sample of 428 patients with chronic diseases, privacy concerns were negatively associated with self-reported health information sharing intention, while ATT, SR, PPE, and TR were positively associated after mutual adjustment. Under the baseline fsQCA specification, low PC appeared in four of five high-intention configurations; PC was omitted and therefore unrestricted in the remaining expression. A truth-table row showed empirical coexistence of high PC and high intention, but not a positive or compensatory contribution of PC. Low-intention expressions and core assignments were sensitive to alternative minimization and analytical choices. The findings are exploratory, sample-specific, and insufficient to establish temporal causality or the effectiveness of platform interventions.

## Figures and Tables

**Figure 1:**
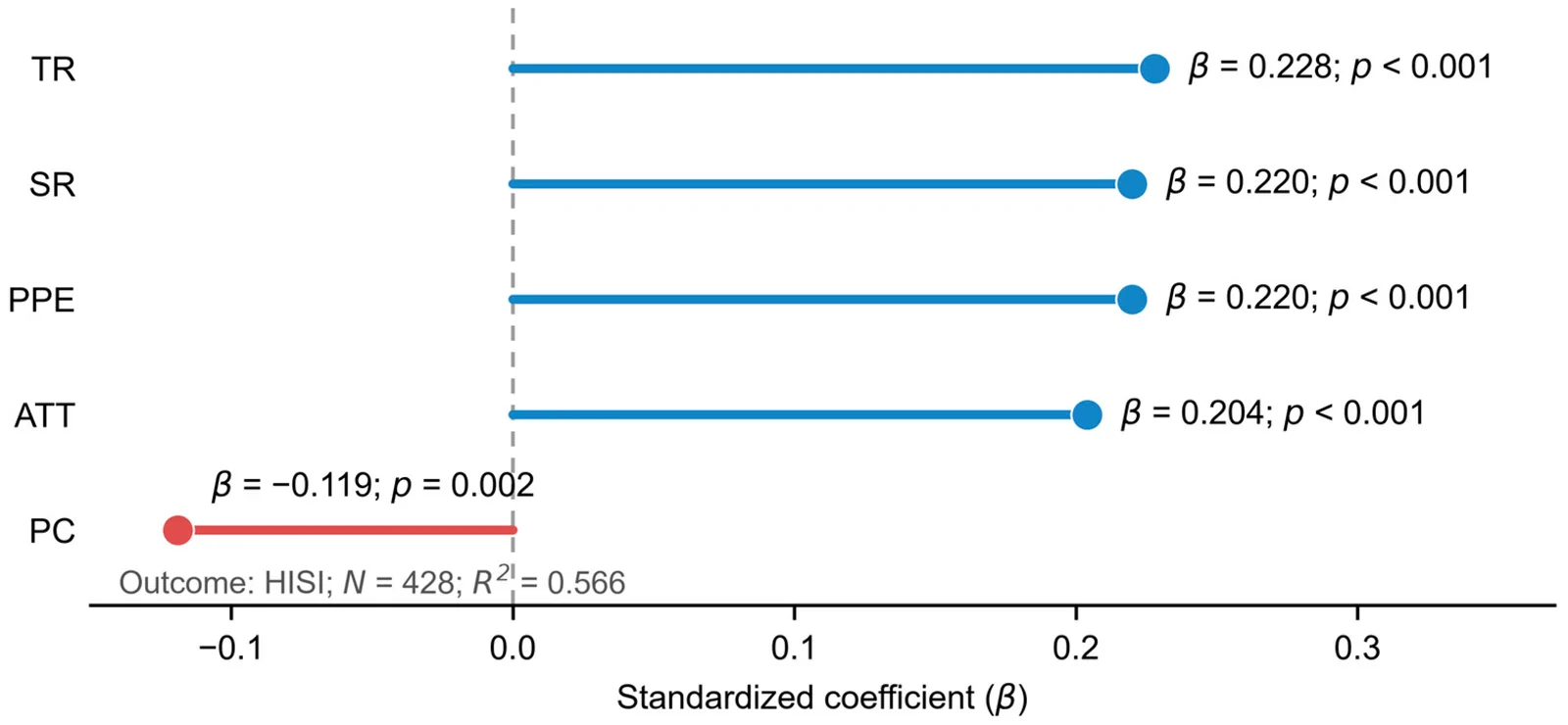
Standardized path coefficients of antecedent constructs and health information sharing intention. Blue represents a positive association and red represents a negative association.

**Table 1:** Correspondence between theoretical propositions and empirical tests.

Proposition	Theoretical Basis	Empirical Expectation	Analysis Method
H1	Theory of planned behavior	ATT is positively related to HISI	SEM
H2	Social benefit mechanism	SR is positively related to HISI	SEM
H3	Experiential learning mechanism	PPE is positively associated with HISI	SEM
H4	Privacy calculus	PC is negatively associated with HISI	SEM
H5	Trust mechanism	TR is positively related to HISI	SEM
RQ1–RQ2	Configurational theory	Multi-condition combination, equivalent path and configurational asymmetry	fsQCA
Post hoc exploratory analysis	Contextual variation	Possible variation in the PC-HISI association; not preregistered or confirmatory	Exploratory interaction analysis

Note: HISI is health information sharing intention; ATT is attitude toward sharing; SR is social rewards; PPE is past positive experience; PC is privacy concerns; TR is trust; SEM is structural equation modeling; and fsQCA is fuzzy-set qualitative comparative analysis.

**Table 2:** Core constructs and operational measurements.

	Code	Number of Items	Operational Content
Health information sharing intention	HISI	3	Sharing intention, disclosing and proactively providing personal health information
Attitude toward sharing	ATT	4	Overall evaluation of whether sharing health information is beneficial, wise, likable, and pleasant
Social rewards	SR	3	Meeting social needs, supporting interpersonal relationships, and gaining psychological satisfaction from interaction
Past positive experience	PPE	3	Previous experiences of receiving help, practical benefits and positive interactions
Privacy concerns	PC	4	Concerns about information misuse, access by others, and unintended use
Trust	TR	4	Evaluation of platform services, demand satisfaction, user benefits and member credibility

**Table 3:** Sample demographics and disease characteristics (N = 428).

Variable	Category	*n*	%
Gender	Male	219	51.2
	Female	209	48.8
Age	18–20 years old	37	8.6
	21–40 years old	105	24.5
	41–60 years old	170	39.7
	Over 60 years old	116	27.1
Educational level	High school and below	94	22.0
	College	102	23.8
	Undergraduate	140	32.7
	Postgraduate degree or above	92	21.5
Internet usage time	1 year or less	28	6.5
	2–4 years	44	10.3
	5–9 years	96	22.4
	10 years and above	260	60.7
Online-health-community usage time	1 year or less	68	15.9
	2–3 years	124	29.0
	4–5 years	154	36.0
	6 years and above	82	19.2
Duration of illness	1 year or less	47	11.0
	2–4 years	95	22.2
	5–9 years	176	41.1
	10 years and above	110	25.7
Chronic-disease type	Cardiovascular disease	189	44.2
	Diabetes	117	27.3
	Chronic obstructive pulmonary disease	85	19.9
	Cancer	34	7.9
	Other	3	0.7

**Table 4:** Descriptive statistics, reliability and convergent validity of core variables.

	Mean	SD	α	CR	AVE	Standardized Factor Loading
HISI	4.623	1.515	0.910	0.910	0.771	0.862–0.894
ATT	4.848	1.375	0.921	0.921	0.745	0.848–0.877
SR	4.818	1.381	0.905	0.906	0.762	0.852–0.903
PPE	4.742	1.420	0.897	0.897	0.744	0.852–0.873
PC	3.274	1.482	0.927	0.927	0.760	0.859–0.886
TR	4.887	1.307	0.908	0.908	0.712	0.816–0.861

Note: SD is the standard deviation, α is Cronbach’s α, CR is the composite reliability, and AVE is the average variance extracted.

**Table 5:** Fit Indices for the measurement and structural models.

Model	χ^2^/df	CFI	TLI	RMSEA	SRMR	χ^2^	df
28-item candidate-set one-factor diagnostic model	12.326	0.617	0.587	0.163	0.137	4314.241	350
Eight-factor CFA diagnostic model	1.219	0.993	0.992	0.023	0.024	392.583	322
Six-factor revised CFA model	1.216	0.995	0.994	0.022	0.025	211.530	174
Six-factor revised structural model	1.216	0.995	0.994	0.022	0.025	211.530	174

Note: The one-factor diagnostic used the 28-item candidate measurement set and is reported only as a common-method diagnostic; its global fit is not directly compared with that of the 21-item revised six-factor model.

**Table 6:** Path estimates of the six-factor main structure model.

Path	b	β	Robust SE	z	*p*	Interpretation	Robust 95% CI for b
ATT → HISI	0.245	0.204	0.068	3.602	<0.001	Direction consistent with H1	[0.112, 0.378]
SR → HISI	0.255	0.220	0.068	3.735	<0.001	Direction consistent with H2	[0.121, 0.389]
PPE → HISI	0.256	0.220	0.071	3.605	<0.001	Direction consistent with H3	[0.117, 0.396]
PC → HISI	−0.125	−0.119	0.041	−3.077	0.002	Direction consistent with H4	[−0.205, −0.045]
TR → HISI	0.277	0.228	0.083	3.355	<0.001	Direction consistent with H5	[0.115, 0.439]

Note: b is the unstandardized coefficient, β is the standardized coefficient, SE is the standard error of the unstandardized coefficient; and HISI’s R^2^ = 0.566.

## Data Availability

The data analyzed in this study were collected through a hospital-based questionnaire survey. Public release of individual-level questionnaire data and calibrated case-level membership scores was not covered by the original participant-consent and institutional-governance arrangements and would be subject to additional ethics and hospital review or approval, as applicable. Aggregated truth tables, calibration rules, analytical settings, solutions, and sensitivity-analysis results are provided in the Supplementary Materials. Requests for access to additional de-identified data may be directed to the corresponding author and will be considered following any applicable review and approval.

## Supplementary Materials

The following supporting information can be downloaded at: https://www.mdpi.com/article/10.3390/healthcare14193337/s1. The following supporting information accompanies this article: Supplementary Files S1–S4 (questionnaire items and sources; descriptive statistics and correlations; complete fsQCA truth table; exploratory interaction and contextual analyses); Supplementary Data S1 (fsQCA: calibration, truth tables, solutions, and sensitivity analyses); Supplementary Data S2 (SEM Model: measurement and structural-model diagnostics, latent-correlation bootstrap confidence intervals, and model fit); and Supplementary Data S3 (Exploratory: post hoc interaction analyses). References [43,44,45,46,47,48,49] are cited in the Supplementary Materials.

## Abbreviations

The following abbreviations are used in this manuscript:

ATT	Attitude toward sharing
AVE	Average variance extracted
CFA	Confirmatory factor analysis
CFI	Comparative fit index
CR	Composite reliability
FDR	False discovery rate
fsQCA	Fuzzy-set qualitative comparative analysis
HISI	Health information sharing intention
HTMT	Heterotrait–monotrait ratio
OHC	Online health community
PC	Privacy concerns
PPE	Past positive experience
PRI	Proportional reduction in inconsistency
RMSEA	Root mean square error of approximation
SEM	Structural equation modeling
SR	Social rewards
SRMR	Standardized root mean square residual
TLI	Tucker–Lewis index
TR	Trust
VIF	Variance inflation factor
