# Supplementary material for: Privacy Concerns and Configurational Patterns of Health Information Sharing Intention Among Patients with Chronic Diseases in Online Health Communities: An SEM and fsQCA Analysis
Source: Healthcare (Basel). 2026 Oct 6;14(19):3337. doi: 10.3390/healthcare14193337 (PMC13665005; doi:10.3390/healthcare14193337)
Supplement: Supplementary file 1 [file healthcare-14-03337-s001.zip › Supplementary_File_S1-S4.pdf]

healthcare

MDPI

Supplementary Materials

Privacy Concerns and Configurational Patterns of Health Information Sharing Intention among Patients with Chronic Diseases in Online Health Communities: An SEM and fsQCA Analysis

File S1-S4

File S1. Questionnaire Items

Table S1. The 28 Items in the Eight-Construct Candidate Questionnaire and Their Sources

Construct	Item	English rendering of item content	Source or adaptation basis
HISI	HISI1	Willingness to share health information with the platform and other users	Ajzen [22]; Xiang et al [13]
HISI	HISI2	Disclosure of health information is normal behavior	Xiang et al [13]
HISI	HISI3	Proactively share health information	Xiang et al [13]
ATT	ATT1	Sharing health information is a good idea	Ajzen [22]; Kim et al [43]
ATT	ATT2	Sharing health information is smart	Ajzen [22]; Kim et al [43]
ATT	ATT3	Like to share health information	Ajzen [22]; Kim et al [43]
ATT	ATT4	Sharing makes people feel happy	Ajzen [22]; Kim et al [43]
PBC	PBC1	I am able to control the health information that I post and share in online health communities	Guo et al. [15], adapted from Kim and Kankanhalli [47]
PBC	PBC2	I can reduce the likelihood of adverse outcomes after posting and sharing health information in online health communities	Guo et al. [15], adapted from Kim and Kankanhalli [47]
PBC	PBC3	I have the necessary knowledge to address problems that may arise after posting and sharing health information	Guo et al. [15]; conceptually consistent with Yi and Davis [48]
PBC	PBC4	I am more willing to share health information when adequate privacy-protection measures are in place	Study-specific contextual item informed by Guo et al. [15], Ajzen [22], and Yi and Davis [48]
SR	SR1	meet social needs	Jiang et al [44]
SR	SR2	promote interpersonal relationships	Jiang et al [44]
SR	SR3	Gain psychological satisfaction and enjoyment	Jiang et al [44]
PPE	PPE1	Communication in the past has been helpful	Song and Zahedi [28]
PPE	PPE2	Benefited more from the community in the past	Song and Zahedi [28]
PPE	PPE3	Frequently engaged in positive interactions in the past	Song and Zahedi [28]
PC	PC1	Worry about health information being misused	Zhu et al [6]
PC	PC2	Worry about others seeing health information	Zhu et al [6]
PC	PC3	Health information is more important than other information	Zhu et al [6]
PC	PC4	Worry about information being used unexpectedly	Zhu et al [6]

Construct	Item	English rendering of item content	Source or adaptation basis
SS	SS1	When I need help with a health problem, someone in the online patient community can offer me suggestions	Tian and Wu [49]
SS	SS2	When I encounter a health problem, someone in the online patient community can provide relevant information to help me overcome it	Tian and Wu [49]
SS	SS3	When I face a health problem, someone in the online patient community can help me identify the cause and offer suggestions	Tian and Wu [49]
TR	TR1	Trust community information and services	Song and Zahedi [28]; Xiang et al [13]
TR	TR2	Communities meet information needs	Song and Zahedi [28]; Xiang et al [13]
TR	TR3	The community cares about the interests of users	Song and Zahedi [28]; Xiang et al [13]
TR	TR4	Other members are trustworthy	Song and Zahedi [28]

Source clarification for candidate-model-only items: Kim and Kankanhalli [47], Yi and Davis [48], and Tian and Wu [49]. Tian and Wu [49] attributed these informational-support items to earlier social-support measures. PBC4 was not found verbatim in these sources and is therefore reported as a study-specific contextual item rather than attributed to an external scale.

Table S2. Operationalization of Exploratory Contextual Variables

variable	Component items/constructs	Indicator type	Scoring	Reliability/Explanation
Information Sensitivity (IS)	Q 15_1 requests health information; Q 15_2 requests other personal information; Q 15_3 requests identity information	Three-item multi-item measure	Arithmetic mean of 7-point Likert items in the same direction	Cronbach's α=0.895
Privacy Sensitivity (PS)	Q 16_1 Medical history; Q 16_2 Medication; Q 16_3 Health status; Q 16_4 Psychological information	Four-item multi-item measure	Arithmetic mean of 7-point Likert items in the same direction	Cronbach's α=0.922
Composite facilitating-factor index	ATT, SR, PPE and TR	Exploratory index composed of four constructs	Arithmetic mean of the standardized construct scores	Not a factor score or a reflective scale; Cronbach's alpha was not calculated

File S2. Descriptive Statistics and Correlation Matrix

Table S3. Pearson Correlation Matrix of Six Construct Composite Scores (N=428)

construct	mean	SD	HISI	ATT	SR	PPE	PC	TR
HISI	4.623	1.515		0.565	0.568	0.558	-0.200	0.560
ATT	4.848	1.375	0.565		0.547	0.530	-0.130	0.543
SR	4.818	1.381	0.568	0.547		0.538	-0.143	0.544
PPE	4.742	1.420	0.558	0.530	0.538		-0.099	0.540
PC	3.274	1.482	-0.200	-0.130	-0.143	-0.099		-0.039
TR	4.887	1.307	0.560	0.543	0.544	0.540	-0.039	

Note: The correlation is based on the average composite score of the items. It is only used for description and does not replace the latent variable structure model. Except for the PC-TR correlation, all other off-diagonal correlations reached $p < 0.05$ in two-sided tests.

Table S4. HTMT Diagnostics for Highly Overlapping Construct Pairs in the Eight-Construct Measurement Model

Construct pair	HTMT	Diagnostic interpretation
PBC-SS	0.976	Exceeds the 0.90 threshold
PBC-TR	0.993	Highest value; exceeds the 0.90 threshold
SS-TR	0.988	Exceeds the 0.90 threshold

Note. N = 428. HTMT values were calculated from the item-level correlation matrix for the candidate eight-construct measurement model. PBC = perceived behavioral control; SS = social support; TR = trust. All three values exceed the 0.90 threshold, indicating inadequate discriminant validity. This local measurement problem is not inconsistent with the good global fit of the eight-factor CFA. The maximum value was observed for PBC-TR (HTMT = 0.993; unrounded value = 0.993259).

File S3. Complete fsQCA Truth Table

Table S5. Truth Table for High and Low HISI (Condition Order: ATT, SR, PPE, PC, TR)

ATT SR PPE PC TR	Frequency	High HISI consistency	High HISI PRI	High results selected	Low HISI consistency	Low HISI PRI	Low results selected
00000	26	0.576	0.108	no	0.940	0.875	yes
00001	0	0.882	0.475	no	0.894	0.525	no
00010	15	0.679	0.111	no	0.948	0.857	yes
00011	4	0.825	0.283	no	0.924	0.687	no
00100	1	0.882	0.501	no	0.882	0.499	no
00101	2	0.901	0.655	no	0.810	0.340	no
00110	3	0.858	0.356	no	0.921	0.644	no
00111	2	0.882	0.553	no	0.854	0.447	no
01000	4	0.894	0.545	no	0.873	0.455	no
01001	1	0.911	0.662	no	0.826	0.338	no
01010	5	0.814	0.280	no	0.926	0.714	yes
01011	1	0.883	0.487	no	0.889	0.513	no
01100	1	0.912	0.677	no	0.816	0.323	no
01101	15	0.906	0.757	yes	0.702	0.227	no
01110	0	0.896	0.505	no	0.894	0.495	no
01111	5	0.891	0.664	no	0.784	0.336	no
10000	0	0.882	0.515	no	0.874	0.485	no
10001	2	0.880	0.611	no	0.812	0.389	no
10010	3	0.869	0.392	no	0.916	0.608	no
10011	1	0.889	0.536	no	0.872	0.464	no
10100	1	0.894	0.648	no	0.805	0.352	no
10101	8	0.902	0.766	yes	0.678	0.229	no
10110	1	0.899	0.559	no	0.873	0.441	no
10111	3	0.896	0.685	no	0.770	0.301	no
11000	1	0.904	0.698	no	0.777	0.302	no
11001	16	0.894	0.762	yes	0.658	0.230	no

ATT SR PPE PC TR	Frequency	High HISI consistency	High HISI PRI	High results selected	Low HISI consistency	Low HISI PRI	Low results selected
11010	1	0.907	0.608	no	0.855	0.392	no
11011	1	0.896	0.673	no	0.780	0.309	no
11100	12	0.918	0.804	yes	0.664	0.196	no
11101	227	0.938	0.917	yes	0.300	0.061	no
11110	3	0.915	0.710	no	0.792	0.290	no
11111	63	0.914	0.853	yes	0.494	0.132	no

Note: 1 indicates a set-membership score >0.5, whereas 0 indicates a score ≤0.5. Main-analysis thresholds were case frequency ≥5, raw consistency ≥0.80, and PRI ≥0.70. No logical remainder was retained as an easy counterfactual in the intermediate minimization under the theory-informed directional expectations used in the reproducibility analysis; these expectations are not described as preregistered. Sensitivity analyses included exact-crossover handling at 0.499, a stricter PRI threshold of 0.75, and calibration, threshold, sample, and item-composition checks. The accompanying aggregate fsQCA workbook reports the truth table, contradictory rows, directional expectations, all solutions, and sensitivity results without case identifiers or case-level membership scores. PRI denotes proportional reduction in inconsistency. Truth tables for high and low HISI were constructed and minimized separately.

File S4. Exploratory Interaction and Contextual Analyses

S4.1 Detailed Analytical Procedure

For the post hoc exploratory interaction analyses, the item-mean composite scores for HISI, ATT, SR, PPE, PC, and TR were standardized across the 428 participants. Each exploratory linear model included all five main effects and one interaction term. The interaction terms were PC × TR, PC × ATT, PC × SR, PC × PPE, and PC × the composite facilitating-factor index. HC3 robust standard errors were used [45]. The Benjamini-Hochberg method controlled the false discovery rate across the five facilitating-condition interaction tests [46]. A three-test contextual family covered PC × information sensitivity, PC × privacy sensitivity, and the omnibus PC × disease-type interaction. A pooled eight-test BH correction was also calculated as a sensitivity analysis. Conditional PC slopes were retained as descriptive model-based associations and were not used to claim significant moderation when the corresponding interaction failed multiplicity correction. Disease type was coded with dummy variables, using cardiovascular disease as the reference group. A joint Wald test with four degrees of freedom assessed all interactions between PC and disease type. The approved protocol permitted exploratory interaction and robustness analyses; however, the particular models, interaction terms, subgroup contrasts, and multiplicity families reported here were not preregistered or prospectively prespecified and were finalized after the primary SEM and fsQCA analyses. They are therefore reported as post hoc exploratory analyses and did not directly account for measurement error in the composite scores.

Information sensitivity (IS) was measured with three items, Q15_1 to Q15_3. They asked about requests from websites or other users for personal health information, other personal information, and identity information. Privacy sensitivity (PS) was measured with four items, Q16_1 to Q16_4. They covered medical history, medication, health status, and psychological information. Both measures used the arithmetic mean of same-direction items on a 7-point Likert scale. Cronbach’s alpha was 0.895 for IS and 0.922 for PS. The composite facilitating-factor index was the arithmetic mean of the standardized construct scores for ATT, SR, PPE, and TR. It was used only to summarize the joint level of the four facilitating conditions. Because these constructs are not interchangeable reflective items, alpha was not calculated for this index. The index was also not treated as a new single construct. Appendix A provides the items and scoring method.

S4.2 Full Results

Table S6. Exploratory Interaction and Contextual Analyses

Effect/level	b or χ^2	HC3 Robust SE	95% CI	p	FDR q/judgment
A. Interaction term test					
PC×TR	0.072	0.032	[0.010, 0.134]	0.022	q=0.055; not significant
PC×ATT	0.021	0.030	[-0.038, 0.080]	0.477	q=0.477; not significant
PC×SR	0.030	0.028	[-0.026, 0.085]	0.296	q=0.370; not significant
PC×PPE	0.076	0.031	[0.015, 0.137]	0.015	q=0.055; not significant
PC × composite facilitating-factor index	0.068	0.034	[0.002, 0.134]	0.044	q=0.073; not significant
PC × information sensitivity	-0.014	0.039	[-0.091, 0.063]	0.721	Contextual-family q=0.721; not significant
PC × privacy sensitivity	-0.047	0.039	[-0.124, 0.030]	0.231	Contextual-family q=0.346; not significant
PC × disease type (overall)	$\chi^2(4)=6.229$			0.183	Contextual-family q=0.346; omnibus interaction not significant
B. PC condition slope					
TR: mean −1 SD	-0.193	0.045	[-0.282, -0.104]	<0.001	Nominal conditional association (p<0.05); interaction not significant after multiplicity correction
TR: mean	-0.121	0.035	[-0.189, -0.052]	0.001	Nominal conditional association (p<0.05); interaction not significant after multiplicity correction
TR: mean + 1 SD	-0.048	0.049	[-0.144, 0.048]	0.323	Not significantly different from zero
PPE: mean −1 SD	-0.192	0.048	[-0.287, -0.098]	<0.001	Nominal conditional association (p<0.05); interaction not significant after multiplicity correction
PPE: Mean	-0.116	0.035	[-0.185, -0.047]	0.001	Nominal conditional association (p<0.05); interaction not significant after multiplicity correction
PPE: mean +1 SD	-0.040	0.046	[-0.130, 0.050]	0.383	Not significantly different from zero

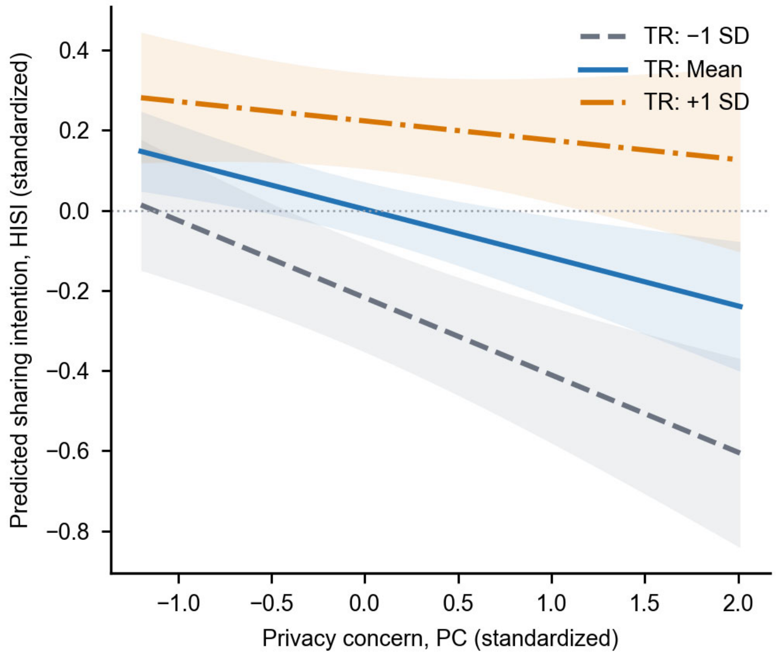

Figure S1. Conditional association between privacy concerns and health information sharing intention at different levels of trust. The lines show model predictions with the other predictors held at their means. The shaded areas show 95% confidence intervals. All variables were standardized.

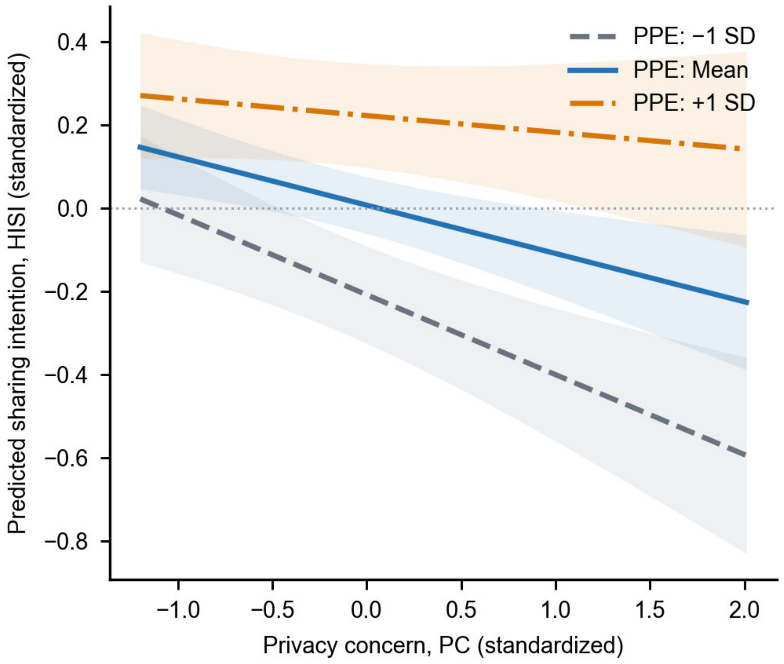

Figure S2. Conditional association between privacy concerns and health information sharing intention at different levels of past positive experience. The lines show model predictions with the other predictors held at their means. The shaded areas show 95% confidence intervals. All variables were standardized.
